# Can hypnosis and virtual reality reduce anxiety, pain and fatigue among patients who undergo cardiac surgery: a randomised controlled trial

**DOI:** 10.1186/s13063-020-4222-6

**Published:** 2020-04-15

**Authors:** Floriane Rousseaux, Marie-Elisabeth Faymonville, Anne-Sophie Nyssen, Nadia Dardenne, Didier Ledoux, Paul B. Massion, Audrey Vanhaudenhuyse

**Affiliations:** 1 Laboratory of Cognitive Ergonomics and Work Intervention, University of Liège, ULiège (B32), Quartier Agora - Place des Orateurs, 2, 4000 Liège, Belgium; 2 Algology Department, University Hospital of Liège, CHU Sart Tilman, Domaine Universitaire du Sart Tilman B35, 4000 Liège, Belgium; 3 Sensation and Perception Research Group, GIGA Consciousness, University of Liège, GIGA (B34), Quartier Hôpital - Avenue de l’Hôpital, 11, 4000 Liège, Belgium; 4 Public Health Department, Biostatistics, University of Liège, CHU (B35), Quartier Hôpital - Avenue de l’Hopital, 11, 4000 Liège, Belgium; 5 Intensive Care Units, University Hospital of Liège, CHU (B35), Quartier Hôpital - Avenue de l’Hopital, 11, 4000 Liège, Belgium; 6 Anesthesia & Intensive care, GIGA Consciousness, University of Liège, GIGA (B34), Quartier Hôpital - Avenue de l’Hôpital, 11, 4000 Liège, Belgium

**Keywords:** Cardiac surgery, Cardiology, Intensive care units, Non-pharmacological approaches, Pain, Anxiety, Fatigue, Hypnosis, Virtual reality, Virtual reality hypnosis

## Abstract

**Background:**

Different non-pharmacological techniques, including hypnosis and virtual reality (VR) are currently used as complementary tools in the treatment of anxiety, acute and chronic pain. A new technique called virtual reality hypnosis (VRH), which encompasses a combination of both tools, is regularly used although its benefits and underlying mechanisms remain unknown to date. With the goal to improve our understanding of VRH combination effects, it is necessary to conduct randomised and controlled research trials in order to understand their clinical interest and potential benefits.

**Methods:**

Patients (n = 100) undergoing cardiac surgery at the Liège University Hospital will be randomly assigned to one of four conditions (control, hypnosis, VR or VRH). Each patient will receive two sessions of one of the techniques: one the day before the surgery and one the day after. Physiological assessments will be made on the monitor and patients will rate their levels of anxiety, fatigue, pain, absorption and dissociation.

**Discussion:**

This study will help to expand knowledge on the application of virtual reality, hypnosis and VRH in the specific context of cardiac and intensive care procedures, and the influence of these non-pharmacological techniques on patient’s anxiety, fatigue, pain and phenomenological experience.

**Trial registration:**

ClinicalTrials.gov: NCT03820700. Date registered on 29 January 2019.

Study recruitment date: October 6, 2018. Study anticipated completion date: December 28, 2020.

## Background

The aim of this study is to better understand the use of non-pharmacological approaches to reduce anxiety among patients in intensive care units (ICUs), and their potential clinical benefits in one specific population of patients, i.e. patients undergoing cardiac surgery.

Anxiety, pain and fatigue are important factors influencing the recovery of patients after surgery. The definition of preoperative anxiety is “an unpleasant state of uneasiness or tension that is secondary to a patient being concerned about a disease, hospitalization, anaesthesia and surgery, or the unknown” [1]. Among patients admitted to hospital for surgery, 20–28% reported high preoperative anxiety and 60% reported minimal anxiety in two studies investigating this issue [2, 3]. Patients’ anxiety before surgery is a risk for postoperative recovery problems and its consequences are of paramount importance in delaying wound healing [4]. In addition, preoperative anxiety is reported to significantly influence the intensity of postoperative pain [5, 6]. Studies have shown that among patients in the ICU, pain is associated with an unpleasant stay, sleep deprivation, increased agitation, high rates of post-traumatic stress disorder and feeling unsafe in the ICU environment [7–13].

Currently, pharmacological treatments for anxiety and pain are well-developed in the ICU environment, where pain and fatigue are most commonly managed by opioid analgesics, propofol and benzodiazepines [14]. Pharmacological treatment could be considered efficient when the patient feels comfortable, with no adverse effects [15]. Yet, opioid analgesics (e.g. morphine) often lead to respiratory sedation, hyperalgesia, depression, nausea, opioid-induced tolerance and dependence [15]. Benzodiazepines can be used in the short term but can lead to strong dependence, with important adverse effects: ataraxia, irritability, nervousness, depression and risk of suicide [16]. Further, some pharmacological interventions (e.g. lorazepam and pregabalin), intended to treat anxiety, fail to decrease preoperative anxiety and postoperative pain [17, 18]. In the ICU environment, deep levels of sedation potentially lead to increased mortality and lengths of stay [19]. In the light of this, non-pharmacological approaches are of interest as complementary techniques to reduce anxiety and pain. Techniques such as hypnosis and virtual reality (VR) have been investigated in numerous studies in the medical field (e.g. algology, oncology, anaesthesia) to reduce pain and anxiety and increase patients’ comfort [20–22]. Hypnosis is defined as a “state of modified consciousness involving focused attention and reduced peripheral awareness, characterized by an enhanced capacity for response to suggestions” [23]. Hypnosis has three main components: absorption, dissociation and suggestibility. Absorption is the tendency to become fully involved in a perceptual, imaginative or ideational experience; dissociation is the mental separation from the environment; and suggestibility is the responsiveness to social cues, leading to an enhanced tendency to comply with instructions and a relative suspension of critical judgment [24]. This technique is considered safe, and one that allows the patient to be focused on his or her inner world, by including cognitive and behavioural components that enable the mind to influence body sensations and perceptions [25–27]. Hypnotic suggestions can be used to modify perception of symptoms such as pain, anxiety and fatigue, in different health-related disorders (e.g. oncology, chronic pain, surgery). In some cases, hypnosis can be a complement to other medication therapy to reduce anxiety before surgery (e.g. presurgical anxiety in coronary artery bypass and cataract surgery) [28–30] and also after surgery (e.g. during weaning from mechanical ventilation) [31]. A recent meta-analysis showed that hypnosis is a highly effective intervention for anxiety and is more effective when combined with other psychological interventions and various clinical applications [28]. Hypnosis is known to reduce acute and chronic pain [32–36] and improve sleep quality [37, 38]. A variety of relaxation techniques have been investigated to improve the quality of sleep in ICU patients (e.g. aromatherapy, earplugs and masks, noise bundle) but results are not convincing in all studies [39, 40]. One review of the literature showed that hypnosis seems to be a promising technique for management of sleep problems; however, more randomised studies are required to support these results [34]. Hypnosis is an efficient treatment in health care, and one that can save time and costs to healthcare providers in some instances [35, 41].

There has been growing interest in the use of virtual reality (VR) in medicine [20]. VR involves computer-generated, immersive and three-dimensional technologies. VR subjective experience is characterized by senses of immersion and presence. Presence refers to the degree to which the subject experiences being in the virtual environment [42, 43], while immersion is the amount of sensory input the VR system creates [44]. Feedback systems with trackers - and often helmet and gloves - allow individuals to be distracted by interacting with a virtual world and make it as “real” as possible [45]. According to Patterson et al. (2006), immersion in VR can isolate the patient from the outside environment and it is effective in distracting the subject’s attention from a painful stimulus [46, 47]. VR has been shown to divert attention from painful stimulation in both highly hypnotizable and less hypnotizable individuals in experimental and clinical settings [48, 49]. VR can also be considered as an efficient non-pharmacological tool to decrease anxiety (e.g. during dental treatment or phobia therapy) [50, 51]. In our experiment, VR is not used during a painful stimulus but before and after a painful and stressful intervention, i.e. surgery. VR distraction is an adjunctive tool to clinical interventions for such issues as acute and chronic pain management, clinical education, cognitive and motor rehabilitation, anxiety management and communication skills training; however, less is known about its efficacy in decreasing fatigue [20, 52].

Virtual reality hypnosis (VRH) is a technique that combines VR hardware/software and hypnotic induction followed by analgesic suggestions [53]. According to Patterson et al. (2004; 2010), “VRH uses a high-resolution, head-mounted display that delivers absorbing visual images and high-fidelity audio that provide an induction […] followed by suggestions for comfort and pain relief” [53, 54]. Studies have demonstrated positive effects of VRH on pain and anxiety [48], but the actual mechanisms of this treatment are not well-known. Hence, it seems that even if immersion is present in VRH and hypnosis, it would not necessarily bring about the same effects as it does with VR distraction. For example, in hypnosis, absorption and dissociation come from the subject who constructs his own world with the hypnotherapist’s suggestions. In VR, that world is imposed on the subject with existing technology. To our knowledge, two randomised studies have previously been conducted to compare hypnosis, VR and VRH in experimental setups [47, 48]. However, there is a need to compare the efficiency of these techniques in clinical practice.

One of the main goals of caregivers is to create the best environment for reducing the patient’s anxiety in surgery and in the ICU by using the patient’s own resources. In this study, we wish to compare three non-pharmacological methods in patients undergoing cardiac surgery and hospitalized in the ICU: hypnosis, VR and VRH. By comparing these non-pharmacological tools, we would be able to better understand their relative efficacy and mechanisms in making the patient more comfortable. Randomised, controlled research trials are necessary to evaluate how the patient’s cognition and perception of these tools can impact the outcomes [20, 26].

## Methods/design

### Aim

The aim of the project is to better understand the impact of VR, hypnosis and VRH on individual perception and sensation in patients hospitalized in cardiac surgery and ICU departments. The primary outcome will be patients’ anxiety levels preoperatively and in postoperative recovery. Secondary assessments will include assessment of pain, fatigue, relaxation, physiological parameters, absorption, dissociation and presence concepts.

### Study registration

This study has been approved by the Ethic Committee of the Faculty of Medicine and the Ethic Committee of the Faculty of Psychology, Speech Therapy and Educational Sciences of the University of Liège. This trial was registered on clinicaltrials.gov with the trial identification number NCT03820700 in January 2019. The trial is currently ongoing and recruiting.

### Eligibility criteria

This study will have a prospective randomised design, and will be a single-centre trial with four arms, including three experimental and one control group. The study sample will comprise adult patients undergoing cardiac surgery (coronary artery bypass graft; mitral heart valve replacement; aortic valve replacement; others). All the study procedures and surgery will be conducted in the University Hospital of Liège (Belgium). Informed consent will be obtained before inclusion of patients.

There will be 100 patients included in the study (25 patients per condition). The participants will be adults undergoing cardiac surgery who have provided informed consent for their participation in the study. This choice is due to the high prevalence of patients undergoing cardiac surgery and accessibility to these patients at the university treatment centre, and the possibility of easily collecting physiological data and patients’ reports. The age of the patients will range from 18 to 90 years. Patients have to be conscious, awake and able to understand and answer in fluent French. Exclusion criteria are psychiatric diseases like dementia, claustrophobia, acrophobia, severe hearing problems, visual impairment or a state of confusion (Table 1).

**Table 1:** Study inclusion and exclusion criteria

Inclusion criteria are:	
- Adults > 18 years of age	
- French-speaking	
- Undergoing cardiac surgery	
- Provision of written consent for their participation	
Exclusion criteria are:	
- Psychiatric diseases	
- Claustrophobia	
- Acrophobia	
- Severe hearing impairment	
- Visual impairment	
- Surgery cancelled or postponed	
Postoperative dropout criteria are:	
- Death during surgery	
- Refusal to continue the study	
- Extreme fatigue	
- Verbal incoherence	
- State of confusion	
- Glasgow Coma Scale [55] score < 14 - Richmond Agitation-Sedation Scale [56] 1 > score < − 1	

### Design

Participants will be randomly included in the following conditions:

1. Control group: daily care only.
2. Hypnosis: taped hypnosis called “Soothing white clouds”.

The hypnosis session will consist of a 20-min hypnosis recording created by M-E Faymonville and A-S Nyssen, both experts in clinical and experimental hypnosis. The recording, named “Soothing white clouds”, includes suggestions about relaxation, positive body sensations and invitation to observe a sunrise and a beautiful landscape, while relaxing in a white cloud chair. Suggestions are focused on variables we wish to improve with the patient (i.e. relaxation) and not on symptom relief. An example of the text (the original text is in French) is as follows: “This experience invites you to discover your resources to find more comfort, calm and healing… I suggest you to find a comfortable position to take full advantage of this moment […] You can discover new perspectives in this soothing white clouds, note others details, and appreciate to be present in this moment […] appreciate the air around you, breathing oxygen, this energy source, which give energy to your body, everywhere it’s needed…”

3. Virtual reality (VR): mountain landscape 3D animation and sounds of nature

For the VR session we will use a head-mounted 3D graphical display with goggles. This VR environment consists of visualization of a landscape accompanied by sounds of nature. The 3D immersive landscape features a shed near a lake at sunrise followed by a relaxing moment in the clouds. This device was constructed by Oncomfort© according to what we wish the patient to visualize in the hypnosis condition (i.e. landscape, sunrise, clouds). The session lasts 20 min and ends on the lake’s edge. There is no verbal suggestion in this VR device. Sounds consist of water sound, birds and the cicada’s song. Participants will not interact with the environment; they are invited to simply watch the 3D animation and relax during the session.

4. Virtual reality hypnosis combination (VRH): mountain landscape 3D animation plus taped hypnosis “Soothing white clouds”.

In VRH, we replace the sounds of nature used for the VR device by the hypnotic tape in order to have the ideal experimental conditions to compare the techniques. The *Soothing white clouds* hypnosis session is combined with a 3D visual movie (immersive landscape and relaxing moment in the clouds (Oncomfort©)), with a duration of 20 min. The text for hypnosis is the same as in the hypnosis group and includes suggestions about relaxation, positive body sensations, invitation to observe a sunrise and a beautiful landscape, while relaxing in a white cloud chair. The hypnosis script was previously recorded (see hypnosis group description) and then integrated into the VR device. In that way, participants can listen to the hypnosis record throughout the VR session.

Initial contact between the investigator and the patient will take place in the patient’s room one day before cardiac surgery. The investigator will request each patient to consent for participation in the study and subsequently record their demographic data (age, gender, surgery type, alcohol and tobacco use). Hypnosis, VR or VRH will be applied in 20 min session: the day before surgery (day − 1 at 5.00 p.m.) and the day after surgery (day + 1 at 2.00 p.m.). Before and after each session, physiological measurements will be recorded (heartbeat, arterial pressure), and a visual analogical scale (VAS) will be used to assess anxiety, fatigue, pain and relaxation. The VAS is a continuous scale subjectively assessed by the patient and ranges from 0 (no pain/anxiety/fatigue/relaxation) to 10 (maximum pain/anxiety/fatigue/relaxation). We ask the patients to assess their “current pain intensity/anxiety/fatigue/relaxation at the moment”. This score determines the intensity of these variables at a given time [57]. After the session, patients will complete questions about absorption and dissociation (Table 2).

**Table 2:** Standard Protocol Items: Recommendation for Interventional Trials (SPIRIT) study schedule

Timepoint	Study period				
	Hospital admission (Day − 1)		Surgery (Day 0)	Intensive care unit admission (Day + 1)	
	***T0***	***T1***		***T2***	***T3***
**Enrolment**					
Eligibility screen	**X**				
Informed consent	**X**				
**Allocation**					
Control, hypnosis, virtual reality, virtual reality hypnosis	**X**				
**Assessments**					
**Baseline:** demographic factors, Dissociative Experience Scale [58]	**X**				
**Primary outcome:** anxiety	**X**	**X**		**X**	**X**
**Secondary variables**: pain, fatigue, relaxation, heart rate, arterial pressure, respiratory rate, oxygen saturation, pupil size	**X**	**X**		**X**	**X**
**Phenomenology scales**: absorption, dissociation, immersion, presence, time perception		**X**			**X**
Nurse’s and patient’s interview		**X**			**X**

### Recruitment and randomisation

**Fig. 1:**
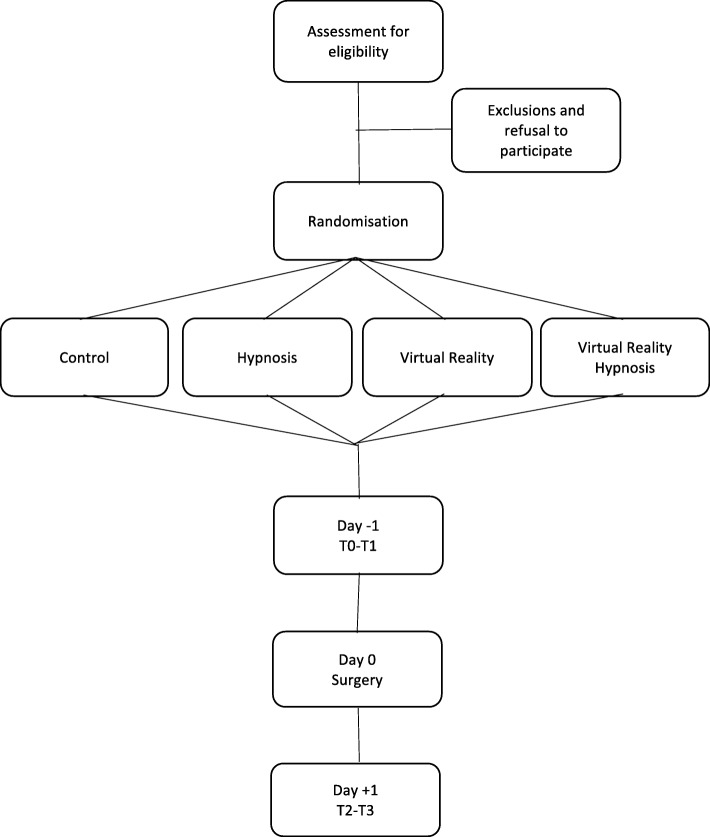
Study flowchart - recruitment and randomisation

Each patient scheduled for cardiac surgery who meets our exclusion and inclusion criteria will be asked to participate. The number of patients who refuse to participate will be recorded, and demographic data will be collected to allow a comparison with those who participate. Written informed consent will be obtained before inclusion. The selected patients will be randomly assigned to one of the four groups, using block randomisation with a block size of 5 to obtain a good balance of participants between the four groups during the recruitment period. The recruitment started in October 2018 and is ongoing. Sample size has been determined by a power analysis calculated to detect a difference in the evolution of data between the four groups. The sample size calculation was based on repeated measures analysis of variance (ANOVA). Alpha was set at 0.05, power at 95% and the standardized effect size at 0.5. In other studies designed to assess the effect of hypnosis on patients’ anxiety pre-surgery, an effect size of 0.2 has been considered small, 0.5 moderate and 0.8 large [29]. According to this analysis, 12 patients are required in each group giving a total of 48 patients. We decided to enrol 100 patients (25 per group) at day 1 to compensate for dropouts on the day after surgery (Fig. 1).

### Assessments

#### Qualitative data

We will record dropouts and the reasons for dropouts. We will record patients’ subjective opinions on hypnosis, VR and VRH, collected through an interview. We also record the nurses’ opinions on hypnosis, VR and VRH collected through an interview about the applicability and the usability of the tools.

#### Demographic factors

We will collect data on age and gender, duration of surgery and type of cardiac surgery (aortic valve replacement, mitral valve replacement or coronary artery bypass surgery). Patients in the ICU are not currently consuming alcohol, but their treatment can be influenced by their previous daily alcohol consumption. The investigator will therefore ask patients about their habitual alcohol and social drug consumption per day and per week. Tobacco withdrawal symptoms (e.g. nervous behaviour) could also influence the participant’s behaviour on the postoperative day. The investigator will therefore ask patients whether they are smokers (yes/no).

#### Psychological outcomes

Anxiety, pain, relaxation and fatigue will be evaluated using a VAS before and after the 20-min sessions. The VAS score helps to determine the intensity of these psychological variables, as subjectively assessed by the patient, on a scale ranging from 0 to 10. The daily dissociative profile will be assessed using the Dissociative Experience Scale (DES) 28-items [58].

Absorption is the “tendency to become fully involved in a perceptual, imaginative, or ideational experience” [59, 60]. We asked subjects to answer this question: “Could you estimate on a 0- (not at all) to 10- (fully) scale how deeply you felt absorbed and felt your attention as focalized and focused by the experience you have just lived?” [60].

Dissociation: is “a mental separation of components of experience that would ordinarily be processed together” [24, 60]. We asked subjects to answer this question: “Could you estimate on a 0-to-10 scale if you felt a dissociation between your bodily sensation and the actual environment? Zero means you were in the reality, in this room; 10 means that you completely escaped in your subjective experience, totally disconnected from the here-and-now reality” [60].

Immersion and presence will be assessed using a VAS. The questions will be “From 0 to 10, how much did you feel present in the environment?” and “From 0 to 10, how much did you really feel the sensations suggested by the therapist?”

Time perception: we will ask subjects to estimate the time elapsed in minutes since they started the session. Time perception will be calculated as the absolute value of the real duration of the experience (hypnosis, VR, VRH) minus the subjects’ estimated duration [60]. The experimenter will record the start and end times of the session.

#### Physiological outcomes

We will assess heart rate: normal heart rate is from 60 to 110 heart beats per minute (depending on whether or not the patient regularly pratices sport) [61]; arterial pressure: normal arterial pressure is from 9.5 to 14.9 [62]; respiratory rate: normal respiratory rate is from 12 to 20 cycles per minute [61]; oxygen saturation: normal values of oxygen saturation are from 95% to 100% [61]; and pupil size: pupil diameter varies from 1 to 10 mm and normal range is from 2 to 8 mm [63].

### Data coding and storage

Data encoding will be assured by the principal investigator of the study (FR). Data will be stored on DOXUlg (https://dox.uliege.be). DOXUlg is a platform for the University of Liège that is secure and confidential. Patients’ data will be accessible only to the principal investigator and promotor to maintain the confidentiality of data.

### Statistical analysis

Normality will be investigated graphically by histogram and quantile-quantile plot and tested using the Shapiro-Wilk test. Continuous variables will be reported as mean (plus/minus standard deviation) or median (interquartile range) for skewed distributions, and qualitative variables as number and percentage. Homogeneity of the four groups will be assessed using the chi-squared test for qualitative and dichotomous variables and one-way (ANOVA-1) or the non-parametric Kruskal-Wallis test for quantitative variables. Repeated measures ANOVA will be used to compare the evolution of the parameters between day − 1 and day + 1 morning and afternoon, according to the groups. This analysis will be adjusted by the potential confounding factors. Calculations are always carried out on the maximum number of data available. Results will be considered as statistically significant at the 5% critical level (*p* < 0.05). Analyses will be performed using R 3.5.3 (R Core Team) and the package Rcommander (Rcmdr) and using SAS 9.4 (© SAS Institute Inc., Cary, NC, USA) [64].

## Discussion

The aim of this study is to evaluate the feasibility of hypnosis, VR and VRH in increasing comfort (anxiety, pain and fatigue) in patients undergoing cardiac procedures, and to investigate the phenomenological experiences they undergo (absorption, dissociation, time perception, immersion and presence). For years, hypnosis and VR have been evaluated in different medical settings and have been shown to be efficient in decreasing perceptions of pain and anxiety [65–68]. More recently, a combination of these two techniques (VRH) was proposed to alleviate clinical symptoms, mainly anxiety and pain [54]. Until now there have been very few controlled studies comparing these techniques [47, 48]. Thereby, our study can potentially make a great contribution in the understanding both of the clinical impact of these approaches and of the mechanisms underlying them. The randomised controlled design is a particular strength of our study. Guidelines are important for tools like VR in terms of mechanisms and clinical benefits. Results of this study will inform us about the endpoint for future well-designed trials forhypnosis, VR and VRH.

There are some limitations to our study. The first limitation could be that some patients will drop out due to inability to participate on the day after surgery. We suspect that extreme fatigue and deep sedation due to surgery may be a barrier to properly following the hypnotic suggestions and the VR animation. The second limitation is that patients are assessed for 2 days and not for the entire period of their hospitalization.

In conclusion, our study will provide initial insight into the application of VR, hypnosis and VRH in the particular context of ICU care, by studying the specific population of patients undergoing cardiac surgery. We will able to measure the effects of VR, hypnosis and VRH on clinically relevant factors such as anxiety and pain. Others studies will then be developed to extend and adapt this protocol to other populations of patients in the ICU.

## Trial status

Trial registration: ClinicalTrials.gov. Registration number: NCT03820700. Date registered: 29 January 2019 Study recruitment date: 6 October 2018. Study anticipated completion date: 28 December 2020. https://clinicaltrials.gov/ct2/show/NCT03820700

## Data Availability

The datasets used and/or analysed during the current study will be available from the corresponding author upon reasonable request.

## Abbreviations

ANOVA: Analysis of variance
ICU: Intensive care unit
VAS: Visual analogue scale
VR: Virtual reality
VRH: Virtual reality hypnosis
